# Multi-drug resistant (MDR) and extended-spectrum β-lactamase (ESBL) producing *Escherichia coli* isolated from slaughtered pigs and slaughterhouse workers in Yaoundé, Cameroon

**DOI:** 10.1016/j.onehlt.2024.100885

**Published:** 2024-08-30

**Authors:** Moise Matakone, Raspail Carrel Founou, Luria Leslie Founou, Brice Davy Dimani, Patrice Landry Koudoum, Marie Christine Fonkoua, Yap Boum-II, Hortense Gonsu, Michel Noubom

**Affiliations:** a Department of Microbiology- Haematology and Immunology, Faculty of Medicine and Pharmaceutical Sciences, University of Dschang, Dschang, Cameroon; b Antimicrobial Resistance and Infectious Disease (ARID) Research Unit, Research Institute of the Centre of Expertise and Biological Diagnostic of Cameroon (CEDBCAM-RI), Yaoundé, Cameroon; c Antimicrobial Research Unit, School of Health Sciences, College of Health Sciences, University of KwaZulu-Natal, Durban 4000, South Africa; d Cameroonian Society for Microbiology, Yaoundé, Cameroon; e Reproductive, Maternal, Newborn and Child Health (ReMARCH) Research Unit, Research Institute of the Centre of Expertise and Biological Diagnostic of Cameroon (CEDBCAM-RI), Yaoundé, Cameroon; f Bioinformatics & Applied Machine Learning Research Unit, EDEN Biosciences Research Institute (EBRI), EDEN Foundation, Yaoundé, Cameroon; g Centre Pasteur du Cameroun, Yaoundé, Cameroon; h Centre Pasteur de Bangui, Bangui, Central African Republic; i Faculty of Medicine and Biomedical Sciences, University of Yaoundé 1, Yaoundé, Cameroon; j University Teaching Hospital of Yaoundé, Yaoundé, Cameroon; k Annex Regional Hospital of Dschang (ARHD), Dschang, Cameroon

**Keywords:** *Escherichia coli*, Multidrug resistance, ESBL, Pigs, Exposed workers

## Abstract

Antimicrobial resistance (AMR) in the food chain remains a global public health concern for both humans and animals. This study aimed to determine the prevalence, resistance profiles, and clonal relatedness of multidrug-resistant (MDR) and extended-spectrum β-lactamases- producing *Escherichia coli* (ESBL-*Ec*) isolated from slaughtered pigs and slaughterhouse workers in Yaoundé, Cameroon.

A cross-sectional study was conducted over four months, from February to May 2023 in two selected pig's slaughterhouse markets in Yaoundé. Rectal swabs were collected from 375 pigs at four time points and pooled per three according to gender, origin, and abattoirs leading to 125 pooled samples. Seven faecal samples from 60 contacted exposed workers were collected. Samples were cultured on CHROMagar™ ESBL medium, dark pink to reddish colonies were considered *E. coli*. Resistance genes including *bla*_CTX-M_, *bla*_SHV_ and *bla*_TEM_ were detected using the polymerase chain reaction (PCR) while ERIC-PCR was used to assess the genetic relatedness between isolates.

The prevalence of ESBL-*Ec* was elevated among exposed workers (71.4 %; *n* = 5/7) and pigs (70.4 %; *n* = 88/125). Overall, ESBL-*Ec* exhibited high resistance to cefuroxime (100 %, *n* = 105/105), cefotaxime (100 %, n = 105/105), amoxicillin-clavulanic acid (98.1 %, *n* = 103/105), cefixime (92.4 %, *n* = 97/105), tetracycline (86.7 %, *n* = 91/105) and sulfamethoxazole-trimethoprim (81.9 %, *n* = 86/105). However, these isolates showed good susceptibility to gentamicin (3.8 %, *n* = 4/105), chloramphenicol (8.6 %, n = 9/105), and fosfomycin (14.3 %, *n* = 15/105)*.* All human isolates and 75.8 % (*n* = 75/99) of pig isolates were multi-drug resistant. The *bla*_CTX-M_ was the most prevalent resistance gene among exposed workers (100 %, *n* = 6/6) and pigs (80.8 %, *n* = 80/99) followed by *bla*_TEM_ (33.3 % each). High clonal relatedness of ESBL-*Ec* strains was observed among pig and human isolates across slaughterhouses.

This study showed that the gastrointestinal tract of pigs might be an important reservoir of MDR and ESBL-*Ec* in Yaoundé, Cameroon and these resistant bacteria might be circulating between sources, especially humans. Heightening awareness on appropriate antibiotic use in humans and animals as well as implementing stringent biosecurity and food safety measures are imperative to prevent the emergence and spread of AMR in the country.

## Highlights

• First detection of humans and pigs faecal carriage sharing ESBL-*E. coli* in Cameroon.
• Identification of highly resistant ESBL-producing *E. coli* among pigs and humans.
• High level of resistance to β-lactams, tetracycline and trimethoprim families.

## 1 Introduction

Antimicrobial resistance (AMR) is a global public health problem that leads to high morbidity and mortality, as well as economic losses, especially in low- and middle-income countries (LMICs) [1,2]. In 2019, there were 4.95 million deaths associated with AMR, with 1.27 million directly attributable to resistant bacteria [3]. Since the last decades, the growing demand for animal protein for human consumption has led to the increasing use of antibiotics in food-animal production [4,5]. The overuse and misuse of antibiotics for growth promotion, treatment and metaphylaxis in food-producing animals have led to the emergence and spread of antibiotic-resistant bacteria throughout the food chain [2,6]. Antimicrobial resistance in food-producing animals affects food safety and security and compromises therefore achievement of the United Nations Sustainable Development Goals 2 and 3 focused on creating a world free of hunger and promoting well-being and healthy lives for all at all ages, respectively, by 2030 [7].

Extended-spectrum β-lactamase-producing *Escherichia coli* (ESBL-*Ec*) remains a major public health issue. Because it is involved in infectious diseases in humans and animals and exhibits a high level of antibiotic resistance especially to β-lactams which are frequently used as frontline drugs to treat infections through the triad of One Health [5,8,9]. *Escherichia coli* is among the foremost pathogens that threaten food security and safety commonly named “foodborne pathogens” [10]. Escalation of foodborne pathogens and AMR across the One Health interfaces are important global challenges. Therefore, the Quadripartite Alliance including the World Health Organization, the World Organization for Animal Health, the Food and Agriculture Organization of the United Nations and the United Nations Environment Program have collectively advocated and supported the establishment of the One Health concept to predict, detect, prevent, and control infectious diseases caused by antimicrobial-resistant pathogens [[11], [12], [13]].

Data are scarce on AMR in sub-Saharan countries (sSA) including Cameroon and lack a robust AMR national surveillance system and infection prevention and control policies [6]. Thus, this study aimed to determine the prevalence, antimicrobial resistance profiles, resistance genes and clonal relatedness of MDR and ESBL-*Ec* isolated from slaughtered pigs and exposed workers in two selected pig slaughterhouses in Yaoundé, Cameroon.

## 2 Materials and methods

### 2.1 Study site and design

**Fig. 1:**
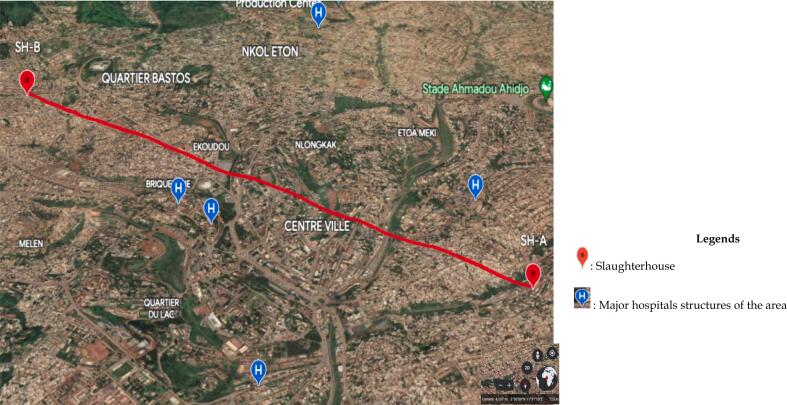
Geographical representation of study sites and surrounding areas.

This cross-sectional study was conducted over four months from February to May 2023 in two selected pig slaughterhouse markets in Yaoundé, the political capital of Cameroon. The central area (centre ville) as illustrated in Fig. 1 contains almost all of the major administrative and hospital structures. Several markets hosting pig slaughterhouses (SH) surround this area. Samples were collected in the two most productive SHs encoded SH-A and SH-B for ethical reasons. The sample analysis was conducted at the Research Institute of the Centre of Expertise for Biological Diagnostic of Cameroon (CEDBCAM-RI).

### 2.2 Sampling and sample collection

In this study, apparently healthy pigs slaughtered during the study period were included. However, dead pigs and those presenting symptoms of illness (confirmed by a veterinarian) or slaughtered out of the abattoir were excluded. The Lorentz formula: n = p x (1-p) x z^2^/d^2^ was used to calculate pigs' sample size considering the prevalence (p) of ESBL-*Ec* from pigs in a previous study in Cameroon estimated at 61 % [14], a confidence level of 95 % (z = 1.96), and 5 % estimated error (d = 0.05), the minimal sample size (n) calculated was 366 pigs. The number of collected pigs was proportional to the annual pig production per site. Rectal samples of freshly slaughtered pigs were obtained by inserting about 3–4 cm of a cotton swab into the anal cavaty and rotating for about 10 s. Then the swab was introduced into Amies transport medium (Copan Italia Spa, Brescia, Italia).

In humans, all workers above 21 years directly in contact with pigs (slaughterers, retailers of viscera and butchers), asymptomatic or presenting clinical signs of infections and willing to participate were included in the study without sex discrimination. Stool samples of exposed workers were collected into sterile containers. Then, samples were transported in an ice box to CEDBCAM-RI and bacteriological analysis was processed within 3 to 4 h of collection.

### 2.3 Ethical considerations

Ethical approval was obtained from the Centre Regional Ethics Committee for Human Health Research (Ref: F001:63CRERSHC/2023). Additionally, the regional (Ref: 00026/L/MINEPIA/SG/DREPIA-CE) and departmental (Mfoundi division) (Ref: 10/2023/L/MINEPIA/DREPIA-CE/DDEPIA-MFDI) authorisations of the Ministry of Livestock, Fisheries and Animal Industries were granted. Finally, approval to carry out the study was obtained from the CEDBCAM-RI (Ref: 2023/02/015/L/CEDBCAM-RI/DG/DRD). Before collecting human samples, oral and written informed consent was obtained from all participants, and a unique code was attributed to each participant for confidentiality purposes.

### 2.4 Culture, identification, and screening of ESBL producers

Pig samples were pooled per three according to gender, breeding origin, and the slaughterhouse in sterile trypticase soya broth. The pooled samples of pigs and human samples were cultured on CHROMagar™ ESBL medium (CHROMagar, Paris-France) which is a selective medium for ESBL isolates. Dark pink to reddish colonies were considered as a *E. coli* according to the recommendation of the manufacturer instructions. However, there were positive samples with more than one *E. coli* morphotypes.

### 2.5 Antimicrobial susceptibility testing

Antimicrobial susceptibility testing (AST) was performed using the Kirby-Bauer disk diffusion method. A 0.5 McFarland bacterial suspension was prepared from fresh colonies and inoculated onto Muller Hinton agar by swabbing. A panel of 10 antibiotics were evaluated after quality control using susceptible *Escherichia coli* ATCC 25922: amoxicillin-clavulanic acid (20/10 μg), cefuroxime (5 μg), cefotaxime (30 μg), cefixime (5 μg), ciprofloxacin (5 μg), gentamicin (10 μg), tetracycline (30 μg), trimethoprim-sulfamethoxazole (1.25/23.75 μg), chloramphenicol (30 μg), and fosfomycin (200 μg). The interpretation of the diameters of inhibition was done after 18 to 24 h of aerobic incubation at 37 °C using the Clinical and Laboratory Standards Institute (CLSI) [15]. Multi-drug resistance which is the resistance to one or more antibiotics from three or more antibiotic families was also investigated [16].

### 2.6 Detection of resistance genes

Bacterial genomic DNA was extracted using the boiling method as previously described [17]. Conventional PCR was performed using the thermal cycler BIO-RAD T100 (Bio-Rad Laboratories Inc., California, USA). Multiplex PCR was done to detect *bla*_TEM_ and *bla*_CTX-M_ while *bla*_SHV_ was detected by singleplex PCR. The reaction occurred in a final volume of 10 μL consisting of 5 μL DreamTaq green polymerase master mix 2× (Thermo Fischer Scientific, Lithuania), 2.8 μL nuclease-free water, 0.1 μL of each reverse and forward primers [10 μM] (Table S1) and 2 μL of DNA. Amplicons were run on 1.5 % agarose gels (wt/vol) with a 100 bp DNA molecular weight marker (New England Biolabs Inc., Ipswich, MA, USA) in a 90 V electric field for 90 min. The gel was stained in ethidium bromide solution (0.5 μg/mL) for 15 min, and the amplicons were visualized under UV light using a gel documentation system G-BOX chemi XL (Syngene, Cambridge, UK).

### 2.7 Genotypic relatedness of ESBL-Ec

Genomic fingerprinting was carried out using ERIC-1 (5’ATGTAAGCTCCTGGGGATTCAC3′) and ERIC 2 (5’AAGTAAGTGACTGGGGTGAGCG 3’) primers [18]. Reactions occurred in a final volume of 10 μL containing 5 μL DreamTaq Green Polymerase master mix 2× (Thermo Fischer Scientific, Lithuania), 2.8 μL nuclease-free water, 0.1 μL of each primer [100 μM], and 2 μL DNA template and run in a thermal cycler BIO-RAD T100 (Bio-Rad Laboratories Inc., California, USA). The ERIC-PCR protocol implemented included 3 min of initial denaturation at 94 °C, followed by 30 cycles consisting of denaturation at 94 °C for 30 s, annealing at 50 °C for 1 min, extension at 65 °C for 8 min, a final extension at 65 °C for 16 min and final storage at 4 °C. Electrophoresis of amplicons was run on 2 % (wt/vol) agarose gel. ERIC profiles were digitized and analysed using GelJ (version 2.1). The dendogram was generated using the Unweighted Paired Group Mean Algorithm (UPGMA) and Dice similarity coefficient.

### 2.8 Statistical analysis

Statistical analysis was done using IBM SPSS statistics version 29.0.1.0 software. A pooled rectal sample and/or human stool sample were considered positive when at least one growing *E. coli* colony was detected on the CHROMagar™ ESBL medium. The ESBL-*Ec* prevalence was compared between modalities of each variable using a chi-square test in pigs and a *p-value <* 0.05 was considered statistically significant.

## 3 Results

### 3.1 Socio-demographic characteristics of the participants

Out of the 60 exposed workers contacted, 14 were enrolled and seven (SH-A, *n* = 5; SH-B, *n* = 2) finally provided stool samples. All the participants were male and most belonged to the 21–30 age group. Most of the abattoir workers (85.7 %, *n* = 6/7) declared to be always in contact with pigs for a whole week, and 57.1 % (*n* = 4/7) did not use safety precautions during the slaughtering process (Table 1).

**Table 1:** Socio-demographic characteristics of the participants.

Variables	Modalities	Frequency n (%)	MDR and ESBL-*Ec* carriage n (%)
Personal characteristics			
Gender	Male	7 (100)	5 (71.4)
Age group	[21–30]	3 (42.9)	2 (66.7)
	[30–40]	2 (28.6)	0
	[40–50]	1 (14.3)	1 (100)
	[50–60]	2 (28.6)	2 (100)
Matrimonial status	Single	4 (57.1)	2 (50.0)
	Married	3 (42.9)	3 (100)
Educational level	Primary	3 (42.9)	1 (33.3)
	Secondary	4 (57.1)	4 (100)
Average month income In CFA Francs	<36,000	3 (42.9)	1 (33.3)
	36,000–50,000	2 (28.6)	2 (100)
	50,000–75,000	2 (28.6)	2 (100)
	75,000–150,000	1 (9.1)	0


Variable	Modality	Frequency n (%)	MDR and ESBL-*Ec* carriage n (%)
Slaughterhouse related factors			
Principal profession	Intermediary seller of living pigs	1 (14.3)	0
	Pig slaughterer and cleaner	5 (71.4)	4 (80.0)
	Wholesaler of living pigs	1 (14.3)	1(100)
Years of experience in the profession	10–14	3 (42.9)	1 (33.3)
	5–9	2 (28.6)	2 (100)
	>15	2 (28.6)	2 (100)
Abattoir	SH-A	5 (71.4)	4 (80.0)
	SH-B	2 (28.6)	1 (50.0)
Living close to abattoir	No	5 (71.4)	3 (60.0)
	Yes	2 (28.6)	2 (100)
Contact with pigs	Almost always	1 (14.3)	1 (100)
	Always	6 (85.7)	4 (66.7)
Safety precautions used	No	4 (57.1)	2 (50.0)
	Yes^#^	3 (42.9)	3 (100)
Waste elimination	Thrown in environment	6 (85.7)	4 (66.7)
	Used as fertilizer	1 (14.3)	1 (100)

Clinical factors			
Antibiotics use in two past weeks	Yes	4 (57.1)	3 (75.0)
	No	3 (42.9)	2(66.7)

NA: not applicable (only variables with two modalities OR was calculated as we used chi-square test); IC: interval of confidence; # use of at least one of these elements: gloves, mask, work clothes, apron, skullcap, boot, convenient hand washing (means with soap or bleach during 30–45 s), wheelbarrows for transport.

### 3.2 Prevalence of MDR and ESBL-*Ec* faecal carriage in humans

**Fig. 2:**
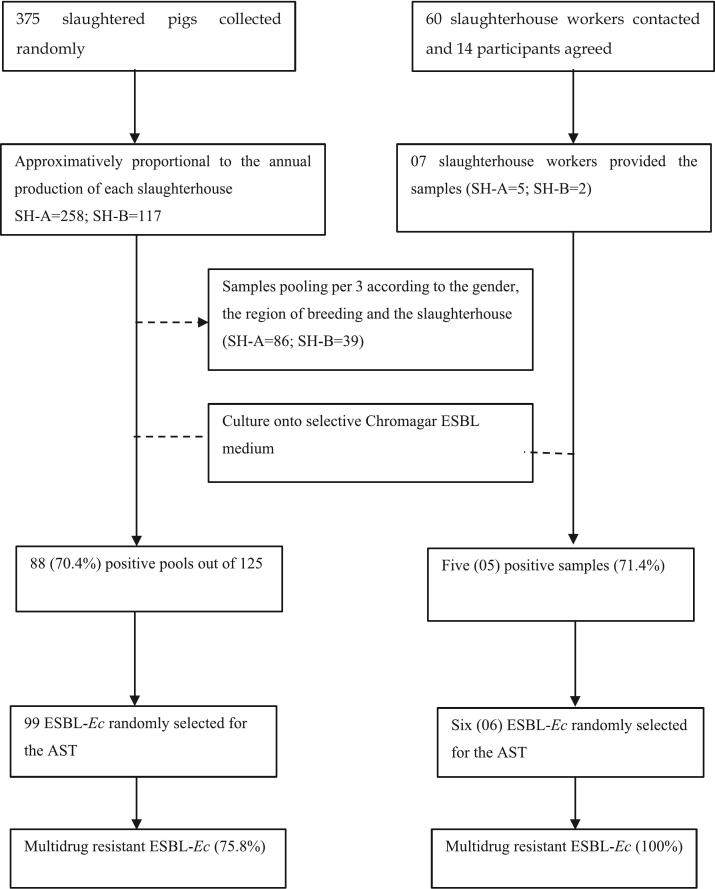
Flowchart of the human and pig isolates. AST: Antimicrobial Susceptibility Testing; ESBL-*Ec*: Extended Spectrum β-Lactamase producing *Escherichia coli*; SH: Slaughterhouse.

The overall prevalence of faecal ESBL-*Ec* carriage was 71.4 % (*n* = 5/7) in exposed workers (Fig. 2). From the five positive cultures, six ESBL-*Ec* were isolated and all were MDR.

### 3.3 MDR and ESBL-*Ec* status in slaughtered pigs

**Fig. 3:**
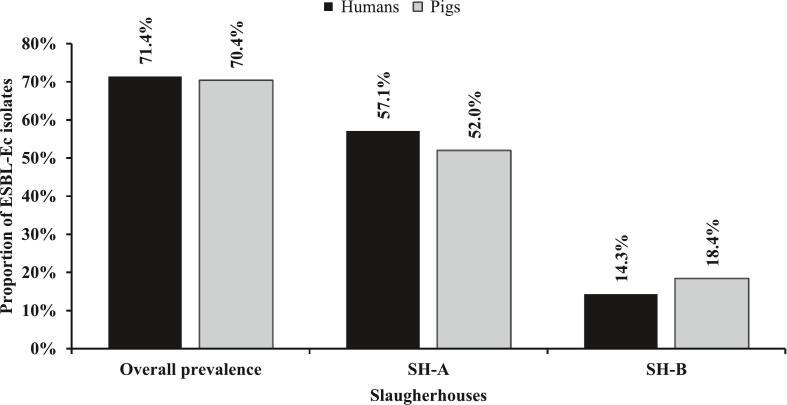
Prevalence of ESBL-*Ec* carriage in humans and pigs across slaughterhouse.

**Fig. 4:**
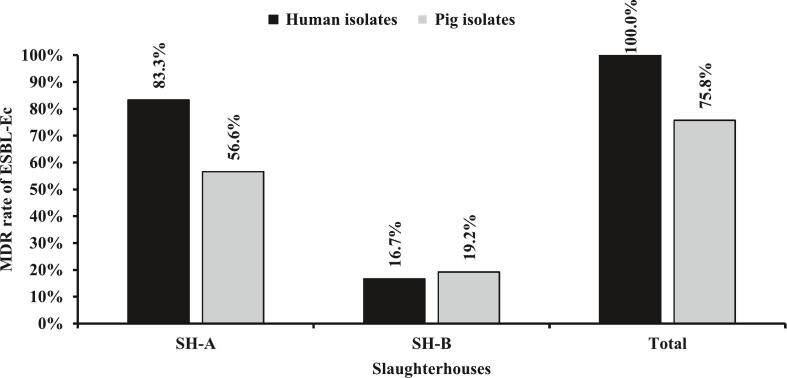
Distribution of multi-drug resistant ESBL-*Ec.*

A total of 375 (SH-A, *n* = 258; SH-B, *n* = 117) non-repetitive rectal swabs were collected and pooled into 125 samples. Slaughtered pigs were principally bred in the Western (55.2 %; *n* = 69/125) and Northern (29.6 %; *n* = 37/125) regions. Out of 125 pooled samples cultured, 70.4 % (*n* = 88/125) were positive for ESBL-*Ec* (Fig. 3) and 52.8 % (*n* = 66/125) yielded at least one MDR isolate. Among the 88 positive pooled samples, 99 ESBL-*Ec* were identified of which, 75.8 % (*n* = 75/99) were MDR (Fig. 4). ESBL-*Ec* carriage was significantly higher among those bred in the Northern region than in the Western region (91.9 %, *n* = 34/37 vs 59.4 %, *n* = 41/69; *p* = 0.001). Table 2 describes the ESBL-*Ec* carriage in slaughtered pigs according to the breeding origin of the slaughtered pig, the gender, the sampling time point, and the abattoir.

**Table 2:** Characteristics of slaughtered pigs.

Variable	Modality	Frequency n (%)	ESBL-*Ec* prevalence n (%)	*p-value*	MDR and ESBL-*Ec* prevalence n (%)	*p-value*
Pig origin	Centre	8 (6.4)	7 (87.5)	**0.002**	05 (62.5)	0.227
	North#	37 (29.6)	34 (91.9)		24 (64.9)	
	North-west	11 (8.8)	6 (54.5)		04 (36.4)	
	West	69 (55.2)	41 (59.4)		33 (47.8)	
Sampling time point	First	15 (12.0)	15 (100)	**< 0.01**	11 (73.3)	**< 0.01**
	Second	33 (26.4)	16 (48.5)		11 (33.3)	
	Third	38 (30.4)	23 (60.5)		15 (39.5)	
	Fourth	39 (31.2)	34 (87.2)		29 (74.3)	
Abattoir	SH-A	86 (68.8)	65 (75.9)	0.06	49 (57.0)	0.165
	SH-B	39 (31.2)	23 (59.0)		17 (43.6)	
Gender	Sow	70 (56.0)	48 (68.6)	0.613	35 (50.0)	0.479
	Boar	55 (44.0)	40 (72.7)		31 (56.4)	

# North: the three northern regions (Adamaoua, North, and Far-North) were grouped together.

### 3.4 Antibiotic resistance profiles of ESBL-*Ec* isolates

Of 105 ESBL*-Ec* isolated, six and 99 were from humans and slaughtered pigs respectively. Overall, ESBL-*Ec* isolates were highly resistant to most of the tested β-lactam antibiotics including cefuroxime (100 %, *n* = 105/105), cefotaxime (100 %, n = 105/105), amoxicillin-clavulanic acid (98.1 %, *n* = 103/105), cefixime (92.4 %, *n* = 97/105), as well as the non β-lactam antibiotics such as tetracycline (86.7 %, *n* = 91/105) and trimethoprim-sulfamethoxazole (81.9 %, *n* = 86/105). Both human and pig isolates showed good susceptibility to gentamicin (3.8 %, *n* = 4/105), chloramphenicol (8.6 %, n = 9/105), and fosfomycin (14.3 %, *n* = 15/105).

In humans, resistance to antibiotics including cefuroxime, cefotaxime, amoxicillin-clavulanic acid, cefixime, tetracycline and trimethoprim-sulfamethoxazole was 100 % (*n* = 6/6) and no resistance was detected against gentamicin. Pig's isolates were mostly resistant to cefuroxime (100 %, *n* = 99/00), cefotaxime (100 %, n = 99/99), amoxicillin-clavulanic acid (98.0 %, *n* = 97/99), cefixime (92.0 %, *n* = 91/99), tetracycline (85.9 %, *n* = 85/99) and trimethoprim-sulfamethoxazole (80.8 %, *n* = 80/99) Table 3.

**Table 3:** Antimicrobial susceptibility test (AST) results of ESBL-*Ec.*

Antibiotics	Resistant isolates n (%)		
	Humans (n = 6)	Pigs (n = 99)	Total (n = 105)
Amoxicillin-clavulanic acid	6 (100)	97 (98.0)	103 (98.1)
Cefotaxime	6 (100)	99 (100)	105 (100)
Ceftazidime	6 (100)	99 (100)	105 (100)
Cefixime	6 (100)	91 (92.0)	97 (92.4)
Cefuroxime	6 (100)	99 (100)	105 (100)
Imipenem	0	0	0
Ciprofloxacin	3 (50.0)	27 (27.3)	30 (28.6)
Gentamicin	0	4 (4.0)	4 (3.8)
Tetracycline	6 (100)	85 (85.9)	91 (86.7)
Fosfomycin	2 (33.4)	13 (13.1)	15 (14.3)
Chloramphenicol	2 (33.3)	7 (7.1)	9 (8.6)
Trimethoprim-sulfamethoxazole	6 (100)	80 (80.8)	86 (81.9)

Four similar antibiotic resistance profiles were identified in human and pig isolates. The AMC-CXM-CTX-CFM-TET-TMP/SXT profile was the most prevalent (37.4, *n* = 37/99, in pigs and 33.3 %, *n* = 2/6 in humans) Table 4.

**Table 4:** Resistant pattern of pig and human ESBL-*Ec* isolates.

Resistance profiles	No. of ESBL-*Ec* in pigs (%)	No. of ESBL-*Ec* in humans (%)	No. of antibiotics family	No. of antibiotics
AMC-CXM-CTX-CFM	7 (7.2)	0	01	4
AMC-CXM-CTX-FF-TET	2 (2.0)	0	03	
AMC-CXM-CTX-CFM-TET	9 (9.2)	0	02	
AMC-CXM-CTX-CFM-TMP/SXT	4 (4.0)	0	02	5
AMC-CXM-CTX-CFM-GEN	1 (1.0)	0	02	
CXM-CTX-CFM-TET-TMP/SXT	1 (1.0)	0	03	
AMC-CXM-CTX-FF-TET-TMP/SXT	4 (4.0)	0	04	
AMC-CXM-CTX-CIP-TET-TMP/SXT	1 (1.0)	0	04	
AMC-CXM-CTX-CFM-TET-TMP/SXT	37 (37.4)	2 (33.3)	03	6
AMC-CXM-CTX-CFM-CIP-TET	1 (1.0)	0	03	
AMC-CXM-CTX-CFM-CIP-TMP/SXT	1 (1.0)	0	03	
CXM-CTX-CFM-CIP-TET-TMP/SXT	1 (1.0)	0	04	
AMC-CXM-CTX-CFM-CIP-TET-TMP/SXT	15 (15.2)	1 (16.7)	04	
AMC-CXM-CTX-CFM-C-TET-TMP/SXT	4 (4.0)	1 (16.7)	04	
AMC-CXM-CTX-CFM-FF-TET-TMP/SXT	3 (3.0)	0	04	7
AMC-CXM-CTX-CFM-GEN-TET-TMP/SXT	1 (1.0)	0	04	
AMC-CXM-CXT-FF-CIP-TET-TMP/SXT	1 (1.0)	0	05	
AMC-CXM-CTX-CFM-C-CIP-TET-TMP/SXT	2 (2.0)	0	05	
AMC-CXM-CTX-CFM-FF-CIP-TET-TMP/SXT	1 (1.0)	2 (33.3)	05	
AMC-CXM-CTX-CFM-GEN-CIP-TET-TMP/SXT	1 (1.0)	0	05	8
AMC-CXM-CTX-C-FF-CIP-TET-TMP/SXT	1 (1.0)	0	06	
AMC-CXM-CTX-CFM-FF-GEN-CIP-TET-TMP/SXT	1 (1.0)	0	06	9
TOTAL	99 (100)	6 (100)	/	/

AMC: Amoxicillin-clavulanic acid; C: Chloramphenicol; CFM: Cefixime; CTX: Cefotaxime; CXM: Cefuroxime; CIP: Ciprofloxacin; FF: Fosfomycin; GEN: Gentamycin; TET: Tetracycline; TMP/SXT: Trimethoprim-sulfamethoxazole.

### 3.5 Distribution of antibiotic resistance genes

**Fig. 5:**
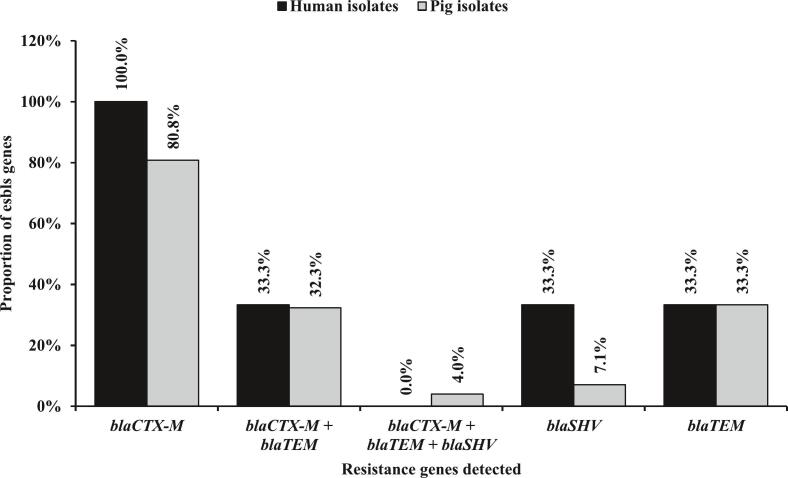
Distribution of resistance genes among ESBL-*Ec* isolated from humans and pigs.

Of the three resistance genes tested, the *bla*_CTX-M_ gene was the most prevalent in both human (100 %, *n* = 6/6) and pig (80.8 %, n = 80/99) isolates followed by the *bla*_TEM_ gene 33.3 % each (n = 2/6 in humans and *n* = 33/99 in pigs). In addition, the prevalence of *bla*_TEM_ was 33.3 % each (n = 2/6 in humans and n = 33/99 in pigs) whereas the *bla*_SHV_ gene was low among pigs (7.1 %, *n* = 7/99) and humans (33.3 %, n = 2/6) isolates. A pig (32.3 %, *n* = 32/99) and human isolates (33.3 %, n = 2/6) harboured concomitantly the *bla*_CTX-M_ and *bla*_TEM_ genes (Fig. 5).

### 3.6 Genetic relatedness of ESBL-Ec isolated from humans and pigs

**Fig. 6:**
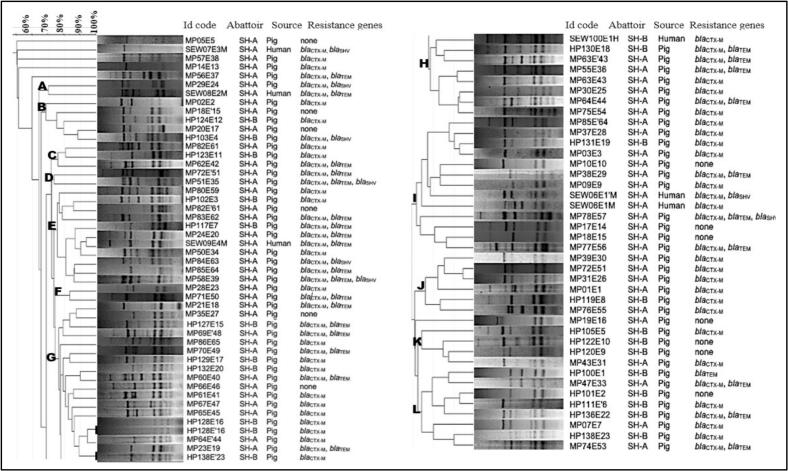
Genetic relatedness of ESBL-*E. coli* (n = 88) isolated from pigs (n = 82) and exposed workers (*n* = 06) in Cameroon. Dendogram generated by GelJ software (version 2.1) using UPGMA and Dice similarity coefficient. The clusters were obtained by similarities of the ERIC genes according to the same genetic pools.

ERIC-PCR was used to evidence the cross-transmission between humans and pigs. The ESBL-*Ec* isolates with at least 80 % similarity were considered to be closely related. Overall, humans (n = 6) and pigs (*n* = 82) ESBL-*Ec* isolates were grouped into 12 clusters designated alphabetically from A to L as indicated in Fig. 6. The cluster E consisting of human and pig isolates from SH-A (MP80E59, MP82E’61, MP83E62, MP24E20, SEW09E4M, MP50E34, MP84E63, MP8564, and MP58E39) and pig isolates from SH-B (HP102E3, HP117E7) were considered to be closely related. Additionally, two pig isolates from SH-A (MP23E19) and SH-B (HP138E’23) were 100 % similar in cluster G.

## 4 Discussion

ESBL-*Ec* is a critical priority pathogen and is transmissible via the food chain causing a public health concern globally [19,20]. The objective of this study was to determine the prevalence, antimicrobial resistance profiles, resistance genes and clonal relatedness of MDR and ESBL-*Ec* isolated from slaughtered pigs and exposed workers in two selected slaughterhouses in Yaoundé, Cameroon.

The prevalence of faecal carriage of ESBL-*Ec* in slaughtered pigs was 70.4 %. A comparable prevalence was recorded in Cameroon in the rectal samples of slaughtered pigs (71 %) [14]. The ESBL-*Ec* carriage in pigs might be correlated to the quantities of antimicrobials, especially β-lactam antibiotics used in farms as a growth promoter by each country [19]. Mouiche et al. (2020) reported that 217.67 t of antimicrobials (by weight of active substance) were imported for veterinary use and tetracyclines (31.17 %), sulfonamides (23.84 %), quinolones (11.11 %) and β-lactams (10.17 %) being the most commonly imported classes of antimicrobials between 2014 and 2019 in Cameroon [21].

The overall prevalence of faecal carriage of ESBL-*Ec* in exposed workers was 71.4 %. The prevalence of ESBL-*Ec* carriage in humans is similar to that found by Godonou et al. (2020) in abattoirs in Lomé, where ESBL-producing *Enterobacteriaceae* were found in the faeces of 80 % of slaughterhouse workers, 78.2 % of which were ESBL-*Ec* [22]. This finding is higher than that demonstrated by Egbule et al. (2020), who reported a prevalence of 50 % of ESBL-*Ec* from pig handlers in retail shops and abattoirs in Nigeria [23]. The observed difference in ESBL-*Ec* carriage might be due to the small human sample size in this study. Nevertheless, the high carriage of ESBL-*Ec* among slaughterhouse workers in African countries could be explained by the fact that hygiene and protection measures during the slaughtering process are not always respected, particularly in our context where access to water is suboptimal [14,24].

The resistance rate was elevated specifically to β-lactams antibiotics and other classes in both human and pig isolates. In addition, 100 % and 75.8 % of human and pig isolates were MDR, respectively. The high resistance level of these isolates to β-lactams and tetracycline families in the present study suggest the overuse of these antibiotic families in the farms and abattoirs before selling. This was supported by Tebug et al. (2020) who recorded that the most prescribed and administered antimicrobials were tetracycline (66 %) followed by β-lactams (32 %) in 20 sub-Saharan African countries [5]. Thus, the use of antimicrobials in animal production should be regulated especially in Sub-Saharan countries including Cameroon.

Also, our finding revealed a high prevalence of ESBLs genes with the *bla*_CTX-M_ being the most prevalent (100 % in humans and 80.8 % in pigs) followed by the *bla*_TEM_ gene (33.3 % for each population). Similar results were documented by Falgenhauer et al. (2018) in Ghana where 96 % and 97 % of *bla*_CTX-M__−__15_ were detected in ESBL-*Ec* isolated from broilers and humans, respectively [25]. The study carried out by Eltai et al. (2020) revealed that 88.23 % of ESBL-*Ec* harboured *bla*_CTX-M_ group 1 (*bla*_CTX-M-15,_ *bla*_CTX-M-3_) and were associated with acute gastro-enteritis among hospitalized children in Qatar [26]. The *bla*_CTX-M -15_ (55 %) was the predominant resistant gene among ESBL-*Ec* isolated from pigs and abattoir workers in the study conducted in slaughterhouse markets in Cameroon [14]. These findings confirm the predominance and active circulation of *bla*_CTX-M_ among humans and animals in Yaoundé, Cameroon. In addition, the national action plan (NAP) on antimicrobial resistance should be strictly implemented in farms and abattoirs respectively and a new strategy needs to be designed to counteract the threat.

Several ESBL-*Ec* isolated from humans were highly similar to those isolated from the slaughtered pigs in both slaughterhouses. The cross-transmission of ESBL-*Ec* between pigs and abattoir workers was previously hypothesised in Cameroon by Founou et al. (2019) [14]. The authors concluded that suboptimal biosecurity and food safety measures observed during the slaughtering process could be a risk factor for the emergence and spread of ESBL-*Ec*.

Despite the high prevalence of ESBL-*Ec* as well as likely cross-transmission among abattoir workers and slaughtered pigs, it is difficult to certify the source of ESBL-*Ec* in this study because pigs from different regions often stay several days in the same pens while waiting to be slaughtered. Thus, these isolates might come from the slaughterhouse environment which is yet to be adequately investigated in the country. Further studies are urgently needed across the farm-to-fork continuum to better understand the epidemiology and transmission dynamics of these resistant bacteria as well as implement tailored prevention and control interventions in the country.

The study has several limitations. First, the small sample areas and limited human sample size did not permit the assessment of the potential risk factors associated with the MDR-ESBL-*Ec* carriage. Second, the number of slaughterhouses considered precludes any generalisation of the results to the country level. Third, few ESBLs genes and no genes conferring resistance to tetracycline, sulfamethoxazole-trimethoprim and amoxycillin and clavulanic acid were investigated which represent antibiotics commonly prescribed for prophylaxis purpose. Fourth, the sequence type (ST) of ESBL-*Ec* circulating between humans and the slaughtered pigs in Yaounde abattoirs could not be ascertained due to financial constraints. Finally, environment samples were not collected and hence, it was not possible to fully elucidate the reservoirs of AMR in the abattoir settings. Notwithstanding, the study generated important baseline data that can be used to invigorate better studies and prevention measures in the food chain.

## 5 Conclusion

The study revealed a high prevalence of MDR and ESBL-*Ec* carriage with non-negligible antibiotic resistance rates in both humans and pigs in Yaoundé, Cameroon. The study also showed a genetic similarity of ESBL-*Ec* isolated in the faecal samples of humans and pigs. These results underscore the need for the implementation of the One Health approach to contain the emergence and spread of AMR in the farm-to-fork continuum in Cameroon.

## Funding

Dr. Raspail Founou received funding from the Merieux Foundation, Lyon, France. This work was supported by the Research Institute of the Centre of Expertise and Biological Diagnostic of Cameroon (CEDBCAM-RI) under the CAREFOOD project. Dr. Luria Founou received funding from the 10.13039/100005627Thrasher Research Fund through the Thrasher Early Career Award (Award number 01364). The funders had no role in the study design, nor the decision to submit the work for publication. Any opinions, findings conclusions or recommendations expressed in this material are those of the author(s) and do not necessarily reflect the views of the organizations or agencies that provided support for the project.

## CRediT authorship contribution statement

**Moise Matakone:** Conceptualization, Methodology, Data curation, Formal analysis, Writing – original draft. **Raspail Carrel Founou:** Funding acquisition, Conceptualization, Methodology, Supervision, Validation, Writing – review & editing. **Luria Leslie Founou:** Funding acquisition, Conceptualization, Writing – review & editing. **Brice Davy Dimani:** Methodology, Formal analysis, Validation, Writing – review & editing. **Patrice Landry Koudoum:** Formal analysis, Writing – review & editing. **Marie Christine Fonkoua:** Validation. **Yap Boum-II:** Validation. **Hortense Gonsu:** Validation. **Michel Noubom:** Supervision, Validation, Writing – review & editing.

## Declaration of competing interest

The authors declare that no conflict could be construed as financial interest.

## Data Availability

All data generated during this study are included in the article.

## Appendix A Supplementary data

Supplementary material: List of primers Image 1
