# Supplementary material for: Multi-drug resistant (MDR) and extended-spectrum β-lactamase (ESBL) producing Escherichia coli isolated from slaughtered pigs and slaughterhouse workers in Yaoundé, Cameroon
Source: One Health. 2024 Aug 30;19:100885. doi: 10.1016/j.onehlt.2024.100885 (PMC11402427; doi:10.1016/j.onehlt.2024.100885)
Supplement: Supplementary file 1 — Supplementary material: List of primers [file mmc1.docx]

| Target gene | Name of primers | Sequence (5’-3’) | Amplicon size (bp) | T° C | References |
| --- | --- | --- | --- | --- | --- |
| TEM | TEM-F  TEM-R | CATTTCCGTGTCGCCCTTATTC  CCAATGCTTAATCAGTGAGGC | 846 | 46.9 | Zemtsa et al., 2022 |
| CTX-M | CTX-MU F  CTX-MU R | CGATGTGCAGTACCAGTAA  TTAGTGACCAGAATCAGCGG | 585 | 46.9 |  |
| SHV | SHV-F  SHV-R | AGCCGCTTGAGCAAATTAAAC  GTTGCCAGTGCTCGATCAGC | 786 | 46.9 |  |
| ERIC | ERIC-1  ERIC-2 | ATGTAAGCTCCTGGGGATTCAC  AAGTAAGTACTGGGGTGAGCG | / | 50 | Versalovic et al., 1991 |

**Table S1:** List of primers
